# Factor XI, a potential target for anticoagulation therapy for venous thromboembolism

**DOI:** 10.3389/fcvm.2022.975767

**Published:** 2022-10-31

**Authors:** Tingting Li, Jiang Liu, Weihua Wu

**Affiliations:** ^1^National Center for Clinical Laboratories, Institute of Geriatric Medicine, Chinese Academy of Medical Sciences, Beijing Hospital, National Center of Gerontology, Beijing, China; ^2^Chinese Academy of Medical Sciences and Peking Union Medical College, Beijing, China; ^3^Department of Nephrology, Metabolic Vascular Disease Key Laboratory, Sichuan Clinical Research Center for Nephropathy, The Affiliated Hospital of Southwest Medical University, Luzhou, China

**Keywords:** abelacimab, anticoagulation therapy, direct oral anticoagulants, factor XI inhibitors, venous thromboembolism

## Abstract

Venous thromboembolism (VTE) is a common cause of mortality and disability in hospitalized patients, and anticoagulation is an essential therapeutic option. Despite the increasing use of direct oral anticoagulants, complications and adverse drug reactions still occur in patients with VTE. Within 5 years, 20% of patients with VTE experience recurrence, and 50% of patients with deep vein thrombosis develop post-thrombotic syndrome. Furthermore, bleeding due to anticoagulants is a side effect that must be addressed. Therefore, safer and more effective anticoagulant strategies with higher patient compliance are urgently needed. Available epidemiological evidence and animal studies have shown that factor XI (FXI) inhibitors can reduce thrombus size and loosen the thrombus structure with a relatively low risk of bleeding, suggesting that FXI has an important role in thrombus stabilization and is a safer target for anticoagulation. Recent clinical trial data have also shown that FXI inhibitors are as effective as enoxaparin and apixaban in preventing VTE, but with a significantly lower incidence of bleeding. Furthermore, FXI inhibitors can be administered daily or monthly; therefore, the monitoring interval can be longer. Additionally, FXI inhibitors can prolong the activated partial thromboplastin time without affecting prothrombin time, which is an easy and common test used in clinical testing, providing a cost-effective monitoring routine for patients. Consequently, the inhibition of FXI may be an effective strategy for the prevention and treatment of VTE. Enormous progress has been made in the research strategies for FXI inhibitors, with abelacimab already in phase III clinical trials and most other inhibitors in phase I or II trials. In this review, we discuss the challenges of VTE therapy, briefly describe the structure and function of FXI, summarize the latest FXI/activated FXI (FXIa) inhibitor strategies, and summarize the latest developments in clinical trials of FXI/FXIa inhibitors.

## Introduction

Venous thromboembolism (VTE) is a common condition in which abnormal blood clotting completely or incompletely blocks blood vessels, impairing the venous blood return. This disorder includes deep vein thrombosis (DVT) and pulmonary embolism (PE). VTE is a leading cause of disability, contributes to the global healthcare burden (1), and is the third leading cause of vascular mortality worldwide, after heart attack and stroke (2). There are many risk factors for VTE, such as genetic factors (including congenital coagulation factor abnormalities or congenital anticoagulant protein defects), and acquired factors like surgery, infection, cancer, and pregnancy (3). However, these risk factors differ according to socioeconomic and health statuses (4).

The current basis of VTE treatment is pharmacological anticoagulation. By using anticoagulants, we have made great progress in recent years in the prevention and treatment of thrombosis, but anticoagulation is still inadequate in clinical practice. Some studies found that the recurrence rate of thrombosis was up to 20% after 5 years in patients with VTE (5), and the estimated mortality rate of patients with PE and DVT after 30 days was 12 and 6%, respectively (6). However, the incidence of anticoagulation-related bleeding is the most common adverse reaction after clinical use of anticoagulants with the incidence of major bleeding per 100 person-years was 1.74 events (7). This is closely related to individual differences and standardized anticoagulation. Patients with advanced age, liver disease, renal insufficiency, bleeding history, cancer and those using antiplatelet drugs, non-steroidal anti-inflammatory drugs, diltiazem, as well as other specific features have a higher incidence of bleeding, and the optimal anticoagulation strategy still needs more research (7–9). Furthermore, the choices of anticoagulant and dose, switch between anticoagulants and bridging therapy strategies remain tricky. The guideline recommends that patients at high risk of thrombosis should be treated with bridging therapy, but the recommendation level is low (10). Recent evidence suggests that bridging therapy leads to a higher incidence of bleeding (11, 12). There are also side effects caused by the drug itself that deserve attention, such as nausea, vomiting and other gastrointestinal reactions caused by direct drug irritation of the gastric mucosa; drug-induced allergic reactions such as asthma, urticaria, and shock; impaired liver and kidney function; osteoporosis; heparin-induced thrombocytopenia (13). Besides, patient compliance and the lack and irrational use of monitoring indicators affect the efficacy of anticoagulant drugs. Moreover, it is urgent and important to find new anticoagulant drugs with milder anticoagulant effects, fewer side effects, and better patient compliance.

There are three predisposing factors for thrombosis—vascular wall damage, altered blood flow, and blood hypercoagulability (14). Damaged vessel walls and endothelial cells can release tissue factor to bind FVII, which then sequentially activates factor X (FX), prothrombin (FII), and fibrinogen (FI) to form thrombi, which is termed the extrinsic coagulation pathway (15–17). The degeneration, necrosis, and shedding of injured endothelial cells expose subendothelial collagen fibers that initiate the intrinsic coagulation pathway by activating factor XII (FXII), which can accelerate clot production (17). In addition, factor IX (FIX) can link the extrinsic and intrinsic coagulation pathways. Therefore, if FXI activity is inhibited, it can inhibit the production of thrombin by the intrinsic coagulation pathway initiated by FXII without affecting the extrinsic coagulation pathway. Thus, FXI, as a link in the intrinsic coagulation pathway, plays an important role in thrombus stabilization and growth, and FXI inhibitors does not seriously affect thrombosis and increase the risk of bleeding.

Research on factor XI (FXI), including genotyping (18–20), clinical data (21–23), and animal studies (24, 25), has shown that high levels of FXI may be a risk factor for VTE. Prolonged activated partial thromboplastin time (aPTT), a thrombus loosening, and reduced thrombus volume can all be caused by genetic abnormalities or reduced activity of FXI. Recent clinical trials have shown the safety and anticoagulant effectiveness of FXI inhibitors. Compared with enoxaparin, abelacimab (26), osocimab (27), milvexian (28), and IONIS-FXI_Rx_ (29) significantly reduced the incidence of VTE and bleeding in patients undergoing total knee arthroplasty (TKA). In clinical studies, osocimab (27) or asundexian (30) showed approximately half the incidence of bleeding events compared with apixaban in the prevention of VTE. Therefore, FXI is considered a novel and safe target for inhibiting thrombosis (31–33). In this review we highlight the urgent need for safer anticoagulants, summarize current strategies for FXI/activated FXI (FXIa) inhibition and clinical trials of FXI/FXIa inhibitors. This information supports the application of FXI/FXIa inhibitors in the prevention and management of VTE.

## Current treatments and challenges of VTE

Anticoagulants, the cornerstone of treatment and prevention of VTE, have evolved from multi-target to single target drugs with better anticoagulation, but still face many challenges in specific situations such as hepatic and renal insufficiency, bridging therapy, combined dosing, safety, and routine monitoring (34, 35). Recent studies have found that FXII and FXI inhibitors single target of intrinsic coagulation pathway and play a limited role in hemostasis *in vivo*, thus they have good anticoagulation effect and safety (36). In addition, FXI inhibitors are metabolized by the liver, but they are tolerated in patients with mild and severe liver damage (37). FXI inhibitors are not only orally available but also maintain anticoagulant effect for up to 1 month (38, 39), which significantly improves patient compliance by eliminating the need for routine monitoring and avoiding the effects of discontinuation or irregular readministration after discontinuation. In addition, FXI inhibitors, regardless of different application strategies, have stable pharmacokinetics, good anticoagulation, and a low incidence of adverse events such as headache and fatigue (37–40). Therefore, FXI inhibitors are safe, but more studies are needed to observe patients with renal insufficiency, combination dosing, and bridging therapy.

Another treatment for VTE is thrombolytic therapy. It is primarily aimed at reducing the burden of thrombosis, but it may not be the most beneficial therapy due to differences in the extent of the disease and thrombolytic regimens (41). Compared to that with anticoagulation alone, pharmacological catheter thrombolysis does not reduce the risk of post-thrombotic syndrome or significantly improve quality of life but increases the risk of major bleeding in patients (42–44). Guidelines on thrombolytic therapy recommend against systemic thrombolysis in patients with DVT, but for those experiencing acute DVT, pharmacological catheter thrombolysis or tissue-type fibrinogen activator can be used (45).

## Structure and function of FXI

FXI was first identified in a family whose main clinical feature was mild to moderate bleeding after tooth extraction (46); the deficient coagulation factor was named plasminogen kinase and the disease was named hemophilia C. FXI is part of the intrinsic pathway and is present in blood circulation mostly as a zymogen. FXI plays an important role in promoting massive production of thrombin and downregulating the fibrinolytic system after the initiation of coagulation (47). FXI can be activated not only by activated FXII (FXIIa) but also by FXIa itself and thrombin in a positive feedback manner; thus, FXI can promote the production of thrombin and amplify the coagulation cascade. However, increased levels of thrombin can increase the concentration of thrombin-activatable fibrinolysis inhibitor, which in turn inhibits the fibrinolytic system and makes the clot more stable.

FXI is mainly synthesized in hepatocytes, along with prothrombin, FXII, and other coagulation factors; it has a distinct structure, which is homologous to that of prekallikrein, although without γ-carboxyglutamate residues, and is a homodimeric protease formed by a disulfide bond linkage (48). The N-terminus of each subunit contains four apple domains (A1–A4), which bind to heparin, FIX, FXIIa, platelet GP1b, and high-molecular-weight kininogen, and are only found in prekallikrein and FXI. The C-terminus is the trypsin-like catalytic domain (49). The structural integrity of FXI is important for thrombosis, but not necessary for hemostasis. Although the monomeric form of FXIa cannot be activated by FXIIa, it can be activated by thrombin or by itself and can even activate FIX in a way similar to the activation of the dimeric form of FXIa (50). The specific mechanism of action and significance of FXI in thrombogenesis are still unclear, and more research is necessary to analyze the mechanism of action of the FXI dimer in thrombosis.

In the presence of polyanions, activators such as FXIIa, thrombin, and FXIa can cause a cleavage of the Arg^369^-Ile^370^ site, which leads to a conformational change of FXI to FXIa (51). Although the structure of FXIa has been described for both subunits of FXI after cleavage of the Arg^369^-Ile^370^ site, some studies have shown that activation of FXI by thrombin or FXIIa to form FXIa is first achieved through the 1/2-FXIa intermediate (52). This intermediate represents a structure generated during FXI activation when only one active subunit has formed after cleavage of the Arg^369^-Ile^370^ site, and its migration rate on the sodium dodecyl sulfate polyacrylamide gel is at the midpoint between that of FXI and FXIa (52). Activation of FIX by FXIa also requires the formation of an activated factor IX (FIXa) intermediate by cleavage at the Arg^145^ site of FIX, which is then converted to FIXaβ. FIXa can then activate FX. This process sustains the early stages of thrombin production, which is further amplified by the action of thrombin on FXI.

In summary, FXI links the thrombin generation system to the kallikrein–kinin system (53). On the one hand, FXIa can activate FXII and FXI, allowing FXI to accelerate the thrombogenic process in the coagulation cascade through positive feedback. However, massive activation of thrombinogen leads to increased activity of the fibrinolytic system. The fibrinolytic inhibitor, which is activated by thrombin, in turn resists the action of the fibrinolytic system and stabilizes the fibrin clot. Thus, FXI is essential not only for contact activation to initiate the coagulation process but also for stabilizing fibrin clots, especially in tissues with high fibrinolytic activity (54).

Hereditary FXI insufficiency is the most common cause of decreases in FXI levels. The *F11* gene in humans is located on the long arm of chromosome 4, and mutations can lead to structural changes in FXI monomers, thereby impairing dimer formation and disrupting FXI secretion. Common mutations include Phe283Leu (50) and Gly350Glu (55) in the A4 domain of the FXI structure, which can lead to a stabilized form of the FXI monomer, thus preventing FXI dimer formation. The mutation of cross-reacting material does not change the structure of the domain but can cause the binding of normal and abnormal subunits to form a non-secretory heterodimer, leading to protein secretion disorder (49). Mutations in residues that bind to ligands, such as thrombin, high-molecular-weight kininogen, heparin, FIX, and platelet GP1b, can also affect the function of the catalytic region of FXIa (56–58). Additionally, severe liver diseases (59) and autoimmune diseases, such as systemic lupus erythematosus (60) and membranoproliferative glomerulonephritis (61), can decrease FXI levels.

Despite the deficiency of FXI, most patients with hemophilia C have an unremarkable clinical presentation and significant individual differences that are usually present during trauma or surgery; these patients are not as prone to spontaneous bleeding or joint bleeding as those with hemophilia A or B (62, 63). Patients experiencing FXI deficiency are more likely to bleed after injury to the gums, sinuses, bladder, endometrium, and other tissues with high endogenous fibrinolytic activity. However, more studies are needed to evaluate the fibrinolytic activity of different tissues and the effect of FXI levels on the occurrence of bleeding after damage to these tissues.

Thus, inhibiting FXI activity can diminish the generation of the fibrinolytic inhibitor, reduce the body's ability to inhibit fibrin clot lysis, and inhibit the intrinsic pathway and amplification of the coagulation cascade. Furthermore, the incidence and severity of bleeding induced by FXI insufficiency are low, suggesting that clinical suppression of FXI activity has a low risk of severe bleeding events and a high level of clinical safety. FXI plays a crucial role in the prevention of VTE and surgical thrombosis, and FXI/FXIa inhibitors are considered promising novel anticoagulants (64).

### FXI/FXIa inhibitors

**Figure 1:**
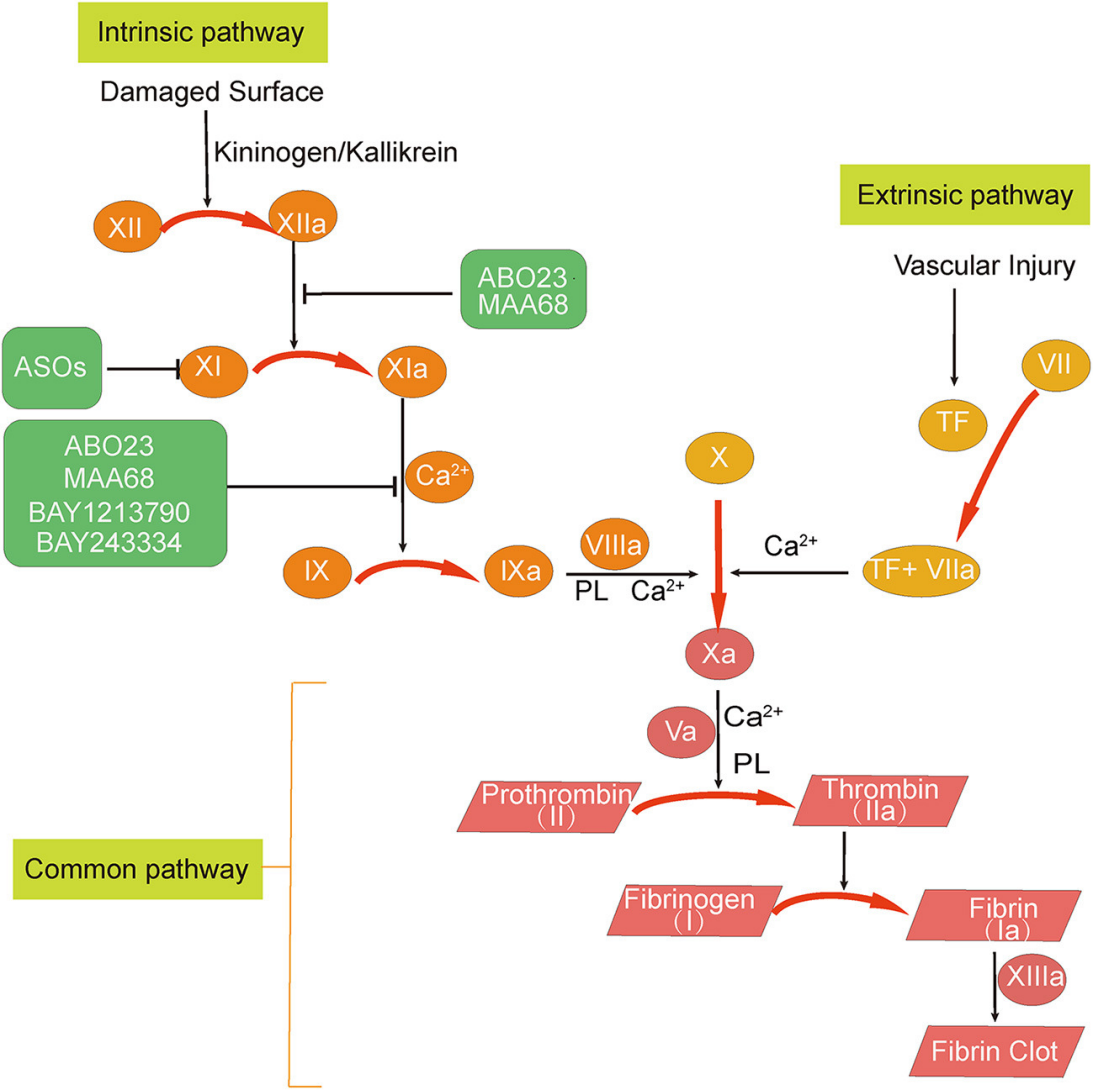
Overview of the sites of action of factor XI (FXI)/activated factor XI (FXIa) inhibitors. The three FXI inhibition strategies can be summarized as follows: (1) degradation of FXI mRNA; (2) inhibition of FXI activation to FXIa; and (3) inhibition of FXIa activity. Abelacimab (MAA868) and osocimab (BAY1213790) are antibodies that inhibit FXIa activity. Abelacimab can also inhibit the activation of FXI by activated factor XII (FXIIa). IONIS-FXIRx and FXI-LICA are antisense oligonucleotides (ASOs) that promote FXI mRNA degradation in the liver and reduce the amount of FXI synthesized. Milvexian (BMS-986177 or JNJ-70033093), asundexian (BAY2433334), SHR2285, and ONO-7684 are small molecules that directly inhibit FXIa. Ir-CPI is a contact-phase peptide inhibitor that can inhibit the activity of activated factor XII and FXIa.

The following sections we describe the main strategies of currently available FXI/FXIa inhibitors. In Table 1 we list the current research strategies for FXI/FXIa inhibitors. In Figure 1 we summarize the current sites of action of the FXI/FXIa inhibitors.

**Table 1:** Current strategies and features of factor XI/activated factor XI inhibitors.

**Type**	**Mechanism of action**	**Delivery**	**Renal clearance**	**Hepatic metabolism**	**Administration**	**Examples**
Antibodies	Bind to FXI/FXIa to inhibit FXI activation and/or FXIa activity	Intravenous or subcutaneous	No	No	Monthly	Osocimab (BAY1213790); Abelacimab (MAA868); AB023 (Xisomab 3G3)
Antisense oligonucleotides	Bind to and catalyze the degradation of FXI mRNA and reduce the hepatic synthesis of FXI	Subcutaneous	No	No	Weekly to monthly	IONIS-FXI_Rx_; FXI-LICA (BAY2976217)
Aptamers	Bind to FXI/FXIa and block their activity	Intravenous or subcutaneous	No	No	Daily	FELIPA
Small molecules	Reversibly bind to the catalytic domain of FXIa and block its activity	Intravenous or oral	Yes	Yes	Daily	Milvexian (BMS-986177/JNJ-70033093); Asundexian (BAY2433334)

FXI, factor XI; FXIa, activated factor XI.

### Antibodies

There are polyclonal and monoclonal antibodies that can inhibit FXI. In animals, FXI is less related to the initiation of thrombosis but is associated with the coagulation process during thrombus formation; therefore, polyclonal anti-human FXI antibodies can reduce the thrombus volume and even lower platelet counts and prolong the aPTT (65). Monoclonal anti-human FXI/FXIa antibodies, such as XI-5108, can dramatically inhibit the thrombus growth by inhibiting substrate binding to FXIa, but they do not inhibit the activation of FXI by thrombin and FXIIa, nor do they inhibit the amidolytic activity of FXIa (66). These antibodies can remarkably prolong the aPTT without affecting the prothrombin time (PT), collagen-induced platelet aggregation, and the bleeding time (66). Monoclonal anti-human FXI antibodies, such as aXIMab, inhibit thrombus development, platelet activation, and thrombin production by binding to the A3 domain of FXI (67). Furthermore, 14E11 (68), DEF (69), and MR1007 (70) have been shown in animal studies to inhibit thrombosis in a dose-dependent manner, without increasing the bleeding time. Unlike other FXIa inhibitors, MR1007 also binds to CD14 to suppress cellular production of interleukin-6 and E-selectin; these anti-inflammatory and anticoagulant functions make MR1007 a viable target drug for treating inflammation-induced hypercoagulable states, such as sepsis and infectious shock (70).

### Antisense oligonucleotides (ASOs)

ASOs are relatively short single-stranded nucleotide sequences that specifically bind to the FXI mRNA in the liver through specific base fragments, resulting in the catalytic degradation of FXI mRNA and reduction of the level of FXI synthesis in the liver (71). Compared with conventional anticoagulants, ASOs have the advantages of lower drug design costs, higher drug targeting selectivity, fewer food and drug interactions, high compliance without frequent doses, and a low incidence of adverse events, such as major bleeding; they are also easy to reverse (71, 72). Studies on ASOs have concluded that they can be used not only to improve hypercoagulable states with thrombotic risk and treat cardiovascular diseases, such as VTE, myocardial infarction, and stroke (73), but also to prevent and treat inflammatory diseases, such as arthritis and colitis (29). However, the backbone of the drug is a phosphodiester skeleton, which is susceptible to the action of nucleases; as this affects the stability and activity of ASOs, it is necessary to modify the backbone to increase its stability during the drug design process (74). Current studies of ASOs include those on IONIS-FXI_Rx_ (29, 75) and FXI-LICA (76).

### Aptamers

Aptamers are single-stranded oligonucleotides obtained through a complex screening mechanism from a DNA combinatorial library, with the advantages of high binding affinity to target proteins, low immunogenicity, low production costs, and the possibility to easily design antidotes (77). The first aptamer that inhibited FXIa was obtained from a database by exponential enrichment to achieve the phylogenetic evolution of the ligand and was finally identified after repeated screening. The inhibitory aptamer can not only inhibit FXIa-mediated FIX activation through competitive inhibition but can also inhibit thrombin production (78). However, this class of drugs does not inhibit the activation of FXI and contains sequences that do not bind to the target. There is also a possibility of the disruption of the aptamer library during the screening process, and there is a need for further optimization. In addition, as limited data exist on the inhibitory abilities of aptamers, more studies are needed to identify drugs with more reasonable structures and clearer efficacy.

### Peptides or peptidomimetics

These drugs can suppress the active site and thus achieve the inhibition by conjugating to the catalytic structural domain. Currently, peptides or peptidomimetics have been developed, including desmolaris (79), protease nexin-2 (80), fasxiator (81), BMS-262084 (82), phenylimidazole (83), α-ketothiazoles (84), and others. Desmolaris, a compound extracted from the salivary glands of *Desmodus rotundus*, can not only inhibit the Kunitz structural domain of FXI and FXIa in a noncompetitive, slow, dose-dependent manner but can also inhibit activated FX, bradykinin, and trypsin to significantly prolong aPTT without producing significant bleeding (79). BMS-262084 can inhibit arterial and venous thrombosis by irreversibly covalently binding to FXIa through the β-lactam structure but cannot significantly prolong the time of bleeding caused by trauma (82). Although there are numerous patents for peptidomimetics (85), related clinical research data are relatively scarce, and more research is necessary to support the pharmacokinetics as well as the safety and efficacy of these drugs in the treatment of different thrombotic diseases.

### Polymeric glycosaminoglycans and saccharide mimetics

Polymeric glycosaminoglycans and their saccharide mimetics are non-competitive conformational inhibitors that are less conservative and more selective than orthosteric inhibitors. The most studied inhibitors are FXIa inhibitors targeting the heparin site (86–88). The mechanism of action may be through the Coulombic attraction of the anionic sulfate group to FXIa cations, followed by the attraction of the heparin-binding site, recognition of the adjacent hydrophobic structure, and tight binding to form a complex (86). This dual strategy ensures that the substance is highly selective for FXIa without inhibiting other proteins, such as thrombin, activated FX, or protein C, which increases the efficacy and safety of the drug. However, the disadvantage of the dual strategy is that it only works for enzymes that bind to both domains; enzymes that lack heparin-binding sites or hydrophobic structures cannot inhibit FXIa. Alternatively, the process can be reversed by fisetin, or the inhibitor activity can be decreased by bovine albumin, FXI, and other substances (87).

## Clinical trials

Through May 20, 2022, a total of 53 registered clinical trials on FXI inhibitors were found in the clinical trials database (https://clinicaltrials.gov/). Only abelacimab has been registered in two phase III clinical trials, where it has been compared with apixaban or dalteparin in the prevention, treatment, and management of tumor-associated VTE. Clinical trials of other FXI inhibitors are in phase I and II and are described in detail below. Table 2 summarizes the clinical trials of FXI/FXIa inhibitors.

**Table 2:** Overview of factor XI-targeting inhibitors currently in clinical trials.

**Compound**	**Therapy**	**Phase**	**Population**	**Number of subjects**	**Comparator**	**Status**	**Registry number**
Abelacimab (MAA868)	Antibody	III	GI/GU cancer-associated VTE	1,020	Dalteparin	Recruiting	NCT05171075
		III	Cancer-associated VTE	1,655	Apixaban	Recruiting	NCT05171049
MK-2060	Antibody	II	ESRD	489	Placebo	Recruiting	NCT05027074
ABO23 (Xisomab 3G3)	Antibody	II	ESRD	27	Placebo	Completed	NCT03612856
		II	Cancer patients on chemotherapy	50	Placebo	Recruiting	NCT04465760
Osocimab (BAY1213790)	Antibody	II	ESRD	686	Placebo	Active, not recruiting	NCT04523220
		II	TKA	813	Enoxaparin or Apixaban	Completed	NCT03276143
BAY2976217 (FXI-LICA)	ASO	II	ESRD	307	Placebo	Active, not recruiting	NCT04534114
IONIS-FXI_Rx_ (BAY2306001/IONIS416858)	ASO	II	ESRD	213	Placebo	Completed	NCT03358030
		II	ESRD	49	Placebo	Completed	NCT02553889
		II	TKA	315	Enoxaparin	Completed	NCT01713361
Asundexian (BAY2433334)	Small molecule	II	AHF	1,592	Placebo	Completed	NCT04304534
		II	Non-cardioembolic ischemic stroke	1,808	Placebo	Completed	NCT04304508
		II	AF	753	Apixaban or Placebo	Completed	NCT04218266
Milvexian (BMS-986177/JNJ-70033093)	Small molecule	II	Stroke	2,366	Placebo	Active, not recruiting	NCT03766581
		II	TKA	1,242	Enoxaparin	Completed	NCT03891524
BMS-986209	Small molecule	II	Healthy participants	114	Placebo	Completed	NCT04154800
ONO-7684	Small molecule	I	Healthy participants	72	Placebo	Completed	NCT03919890
SHR2285	Small molecule	I	TKA	500	Enoxaparin	Recruiting	NCT05203705
Ir-CPI	Polypeptide	I	Healthy males	32	Placebo	Active, not recruiting	NCT04653766

AF, atrial fibrillation; AHF, acute heart failure; ASO, antisense oligonucleotide; GI, gastrointestinal; GU, genitourinary; ESRD, end-stage renal disease; TKA, total knee arthroplasty; VTE, venous thromboembolism.

### Phase III

#### Abelacimab

Abelacimab, previously known as MAA868, is the first FXI/FXIa inhibitor in phase III clinical trials. It is a humanized monoclonal antibody that inhibits the activation of FXI and the activity of FXIa with a lasting inhibitory effect on thrombosis (38, 89). It has the advantages of inhibiting both FXI and FXIa and causing less damage to the liver and kidney, which makes the drug better for use in patients with hepatic and renal dysfunction. Moreover, it has a long half-life and requires injection only once a month, which simplifies the treatment process and improves patient compliance, thus showing good clinical application prospects.

The first phase I clinical trial was conducted with a single incremental dose and showed that a subcutaneous dose of 5 to 240 mg/kg was safe and effective in healthy or obese patients. The prolongation of the aPTT was maintained for at least 4 weeks at a dose of 150 mg/kg, with no significant effect on the PT and thrombin time, and recombinant activated factor VII reversed the effects of MAA868 (38). Intravenous or multiple subcutaneous dosing was also safe in patients with atrial fibrillation (90). A parallel controlled phase II clinical trial of patients who underwent TKA and were randomly administered 30, 75, or 150 mg of intravenous abelacimab or subcutaneously injected with 40 mg of enoxaparin showed that the incidence of VTE was 13, 5, 4, and 22%, respectively, and the incidence of bleeding was 2, 2, 0, and 0%, respectively (26). Two phase III trials, NCT05171049 and NCT05171075, are currently enrolling patients in 2022. NCT05171049 is being conducted at multiple hospitals and is recruiting patients who received direct oral anticoagulants for at least 6 months after a diagnosis of cancer combined with VTE. NCT05171075 aims to compare the efficacy and safety of abelacimab and apixaban to prevent recurrent VTE in patients with gastrointestinal or genitourinary cancers.

### Phase II

#### Milvexian (BMS-986177/JNJ-70033093)

Milvexian, also known as BMS-986177 or JNJ-70033093, is a reversible small-molecule inhibitor of FXIa, with excellent antithrombotic effects, and is also effective in combination with aspirin (91). Currently, milvexian has been registered in the largest number of clinical trials, predominantly phase I clinical trials and two phase II clinical trials. One phase I clinical trial of milvexian was performed on healthy volunteers and showed a promising safety record of the drug (92). The safety of milvexian does not appear to differ depending on race, food interactions, and liver function (37, 93). A pharmacokinetic study observing Milvexian in combination with itraconazole or diltiazem in healthy subjects showed that itraconazole and diltiazem could increase milvexian exposure without affecting the efficacy or increasing the incidence of adverse events (94). This suggests a promising application for the combination of FXI inhibitors with other drugs. Furthermore, there is another observational study on the safety and pharmacodynamics of milvexian in patients with end-stage renal disease on hemodialysis (NCT02902679).

Phase II clinical trials of milvexian included a multicenter study of 923 patients undergoing unilateral TKA who received oral milvexian at a dose of 25, 50, 100, or 200 mg once or twice a day and 296 patients who received 40 mg of enoxaparin once daily (28). In this study, the observed incidence of VTE was 12% in patients taking milvexian and 21% in patients taking enoxaparin. Although the incidence of bleeding was 4%, the occurrence of serious adverse events was 2 and 4% in patients with milvexian or enoxaparin, respectively. Therefore, in patients undergoing TKA, oral milvexian was more effective and safer than enoxaparin in preventing VTE. Another phase II clinical trial (NCT03766581) is a polycentric, randomized, double-blind study. It aims to evaluate the efficacy of secondary stroke prevention with milvexian combined with aspirin and clopidogrel in patients over 40 years of age with acute ischemic stroke or transient cerebral infarction. The primary outcome of the study is the occurrence of a new ischemic stroke or occult cerebral infarction.

#### Osocimab

Osocimab, also known as BAY1213790, is a long-lasting humanized monoclonal antibody against FXIa, which binds to a specific region of the FXIa catalytic structural domain, close to the active site of the enzyme (95, 96). A phase I clinical trial of osocimab was a single-blind study conducted in 83 healthy white men who received single intravenous injections at doses ranging from 0.015 to 10 mg/kg. The results showed no adverse events, and the drug half-life was approximately 30 to 44 days (39). A phase II clinical trial of osocimab was a non-inferiority trial vs. enoxaparin and apixaban that was conducted on 813 patients from 54 hospitals in 13 countries (27). The results indicated that the preoperative use of osocimab at a dose of 0.6 or 1.2 mg/kg has a promising application. There is also an ongoing multicenter phase II clinical trial (NCT04523220), which is being conducted in several countries to investigate the pharmacokinetics and safety of low-dose osocimab (initial load of 105 mg and a monthly maintenance dose of 52.5 mg) and high-dose osocimab (initial load of 210 mg and a monthly maintenance dose of 105 mg) vs. a placebo in patients with end-stage renal failure undergoing regular hemodialysis.

#### ASOs

Currently, the most well-studied ASOs are IONIS-FXI_Rx_ and FXI-LICA. IONIS-FXI_Rx_, also known as BAY2306001 and IONIS416858, inhibits the synthesis of FXI in the liver, which in turn inhibits thrombosis. A phase II clinical trial of this ASO was conducted on a total of 300 patients undergoing TKA in five countries (29). The patients were randomized and received 200 or 300 mg of IONIS-FXI_Rx_ or 40 mg of enoxaparin once daily, and the observed incidence of VTE was 27, 4, and 30%, respectively, whereas the incidence of bleeding was 3, 3, and 8%, respectively. Therefore, the efficacy of 200 mg of IONIS-FXI_Rx_ in the prevention of VTE was not inferior to that of enoxaparin, and the efficacy of 300 mg of IONIS-FXI_Rx_ was better than that of enoxaparin. Other results of phase II clinical trials in patients with end-stage renal disease are in the process of being published (76). FXI-LICA (BAY2976217), also known as ION-957943, has the same RNA sequence as that of IONIS-FXI_Rx_. Based on previous studies, the dose relationship was extrapolated from the IONIS-FXI_Rx_ study to FXI-LICA for validation, and the data showed that 40, 80, and 120 mg of FXI-LICA could be used as doses for clinical trials in patients (76). Therefore, a phase II clinical trial (NCT04534114) is focused on the efficacy and safety of 40, 80, and 120 mg of FXI-LICA in inhibiting of thrombosis in patients with end-stage renal disease undergoing regular hemodialysis, with bleeding as a primary endpoint.

#### AB023

ABO23, also known as xisomab 3G3 or 3G3, is a humanized FXI antibody, but unlike abelacimab and osocimab, it inhibits the activation of FXI by FXIIa rather than inhibiting FXIa activity or activation of FXI by thrombin. The first *in vivo* human study included 21 healthy volunteers and resulted in no positive antidrug antibodies and no serious adverse events at doses of 0.1–5.0 mg/kg (97). A phase II clinical trial of ABO23 (98) was a double-blind trial in 24 patients diagnosed with end-stage renal disease who were administered 0.25 or 0.5 mg/kg AB023 or a placebo. The study demonstrated that ABO23 significantly reduced the occurrence of blood clotting in the dialyzer and was well tolerated by the volunteers. However, the sample size in this study was small. Another phase II clinical trial (NCT04465760) is currently recruiting volunteers and aims to evaluate the effectiveness and safety of ABO23 in preventing central venous catheter-related thrombosis in patients diagnosed with solid tumors treated with peripheral central venous catheter insertion or indwelling. ABO23 may benefit patients using medical devices that come into contact with blood, such as mechanical heart valves or extracorporeal membrane oxygenators (99).

#### Asundexian (BAY2433334)

Asundexian is an orally available, direct small molecule that specifically inhibits FXIa and significantly reduces FXIa activity, prolongs the aPTT, decreases the weight of arteriovenous thrombus, and is independent of antiplatelet drugs (100). The published phase II clinical trial was a double-blind, multicenter study in 753 patients over 45 years of age with atrial fibrillation (30). The volunteers were randomized and administered 20 or 50 mg of asundexian once a day and 5 mg of apixaban twice daily. The final incidence ratios with bleeding as the primary endpoint were 0.50, 0.16, and 0.33, and the incidence of any adverse event was 47, 47, and 49%, respectively. Therefore, 20 or 50 mg of asundexian daily could provide durable inhibition of FXIa and resulted in a lower incidence of bleeding than that with the standard treatment for atrial fibrillation. Two other phase II clinical trials have also been completed, but their results have not yet been published. The primary objective of NCT04304534 was to examine the optimal dose of asundexian in patients with acute myocardial infarction taking acetylsalicylic acid and clopidogrel. The primary outcome was the occurrence of myocardial infarction, stroke, stent thrombosis, and bleeding within 12 months. Another clinical trial, NCT04304508, was also a multicenter, randomized controlled trial. This study was designed to explore the optimal dose of the drug in patients with acute non-cardiogenic stroke.

#### SHR2285

SHR2285 is an oral small-molecule inhibitor of FXIa that can significantly prolong the aPTT and inhibit thrombosis. The main purpose of a phase I clinical trial, NCT03769831 (40), was to evaluate the occurrence of adverse events in healthy volunteers who were administered 50, 100, 200, 400, 600, 800, or 1,000 mg of SHR2285. The results showed that the adverse events involved mainly liver function, gastrointestinal function, and the hematological system, but these recovered spontaneously without any special intervention, and no serious adverse events were observed. In terms of efficacy, SHR2285 could significant prolon aPTT and decrease the activity of FXI, but no significant differences in PT and international standardized ratio were observed. Consequently, SHR2285 performed well in the clinical trial. The follow-up phase II clinical trial (NCT05203705) was a multicenter study that assessed the efficacy and safety of four different doses of SHR2285 vs. enoxaparin to prevent VTE in postoperative patients after unilateral TKA. The primary endpoint of the study was the occurrence of VTE or bleeding after 12 days of treatment.

### Phase I

There are also several FXI inhibitors undergoing phase I clinical trials. ONO-7684, a small molecule inhibitor of FXIa, was first tested in healthy people and showed good safety and tolerability profiles (101). In addition, the half-life of the drug was extended up to 22.1–27.9 h while maintaining a once-daily dosing protocol, suggesting that ONO-7684 is promising for use in anticoagulation therapy. REGN9933 is a monoclonal antibody against FXI, and the NCT05102136 study is expected to enroll 72 healthy adults to evaluate its pharmacokinetics. An *Ixodes ricinus* contact-phase inhibitor (Ir-CPI) is as effective as heparin in inhibiting thrombosis in animal models, but with a reduced incidence of bleeding (102). A phase I clinical trial (NCT04653766) included 32 male volunteers who were randomized and received Ir-CPI at doses of 1.5, 3.0, 6.0, or 9.0 mg/kg; only one serious adverse event was observed at the maximum dose.

## Conclusions

Although the use of direct oral anticoagulants has simplified the prevention and treatment of VTE, the incidence and mortality from VTE are still relatively high, which carries a heavy burden on both patients and healthcare systems. Therefore, there is an urgent need for safer and more effective anticoagulant drugs to prevent VTE. It is also necessary to integrate information on patients' renal function and compliance, medical costs, and contraindications to thrombolysis to assess the risks and benefits of treatment. Since it plays an important role in thrombosis rather than in hemostasis, FXI has received increasing attention as a safer target of anticoagulation. Many FXI/FXIa inhibitors have entered phase II clinical trials, wherea abelacimab is in phase III trials. Completed clinical trials have shown that FXI/FXIa inhibitors are safe and effective in preventing VTE and have broad application prospects, especially in patients with end-stage renal disease undergoing hemodialysis and those with TKA.

More data may be needed to demonstrate the efficacy of FXI/FXIa inhibitors in the prevention and management of VTE in special populations, such as patients with cancer or hepatic and renal insufficiency, children, and older patients. In addition, more data are needed to validate the antagonists of FXI inhibitors and their combinations with other drugs. Indicators for monitoring the drug activity of FXI inhibitors need to be further investigated. More data are needed to establish the correspondence between the doses of FXI inhibitors and aPTT levels, which can be helpful for dose adjustment in clinical treatment. Furthermore, the accuracy of aPTT test results is related to the sensitivity of reagents to drugs, and thus, it is also worth investigating how to avoid the influence of coagulation factors on intrinsic pathways and improve the accuracy and comparability of results from different laboratories. Therefore, more effort is needed to bring FXI/FXIa inhibitors into clinical practice to benefit more patients.

## Author contributions

TL and JL discussed and wrote the manuscript together. WW contributed to the discussion of the content and edited the article before submission. All authors contributed to the article and approved the submitted version.

## Conflict of interest

The authors declare that the research was conducted in the absence of any commercial or financial relationships that could be construed as a potential conflict of interest.

## Publisher's note

All claims expressed in this article are solely those of the authors and do not necessarily represent those of their affiliated organizations, or those of the publisher, the editors and the reviewers. Any product that may be evaluated in this article, or claim that may be made by its manufacturer, is not guaranteed or endorsed by the publisher.
